# Power and Thermal Efficiency Optimization of an Irreversible Steady-Flow Lenoir Cycle

**DOI:** 10.3390/e23040425

**Published:** 2021-04-02

**Authors:** Ruibo Wang, Yanlin Ge, Lingen Chen, Huijun Feng, Zhixiang Wu

**Affiliations:** 1 Institute of Thermal Science and Power Engineering, Wuhan Institute of Technology, Wuhan 430205, China; ruibowq@126.com (R.W.); geyali9@hotmail.com (Y.G.); huijunfeng@139.com (H.F.); zhixiangwuhg@outlook.com (Z.W.); 2 School of Mechanical & Electrical Engineering, Wuhan Institute of Technology, Wuhan 430205, China

**Keywords:** finite time thermodynamics, irreversible Lenoir cycle, cycle power, thermal efficiency, heat conductance distribution, performance optimization

## Abstract

Using finite time thermodynamic theory, an irreversible steady-flow Lenoir cycle model is established, and expressions of power output and thermal efficiency for the model are derived. Through numerical calculations, with the different fixed total heat conductances ($U_{T}$) of two heat exchangers, the maximum powers ($P_{\max}$), the maximum thermal efficiencies ($\eta_{\max}$), and the corresponding optimal heat conductance distribution ratios ($u_{L_{P}(opt)}$) and ($u_{L_{\eta }(opt)}$) are obtained. The effects of the internal irreversibility are analyzed. The results show that, when the heat conductances of the hot- and cold-side heat exchangers are constants, the corresponding power output and thermal efficiency are constant values. When the heat source temperature ratio (τ) and the effectivenesses of the heat exchangers increase, the corresponding power output and thermal efficiency increase. When the heat conductance distributions are the optimal values, the characteristic relationships of $P-u_{L}$ and $\eta -u_{L}$ are parabolic-like ones. When $U_{T}$ is given, with the increase in τ, the $P_{\max}$, $\eta_{\max}$, $u_{L_{P}(opt)}$, and $u_{L_{\eta }(opt)}$ increase. When τ is given, with the increase in $U_{T}$, $P_{\max}$ and $\eta_{\max}$ increase, while $u_{L_{P}(opt)}$ and $u_{L_{\eta }(opt)}$ decrease.

## 1. Introduction

Finite time thermodynamic (FTT) theory [1,2,3,4] has been applied to the performance analysis and optimization of heat engine (HEG) cycles, and fruitful results have been achieved for both reciprocating and steady-flow cycle models. For the steady-flow models, FTT was also termed as finite physical dimensions thermodynamics by Feidt [5,6,7,8,9,10]. The famous thermal efficiency formula $\eta =1-\sqrt{T_{L}/T_{H}}$, where $T_{H}$ and $T_{L}$ are the temperatures of the heat source and heat sink of a HEG, was derived by Moutier [11] in 1872, Cotterill [12] in 1890, and Novikov [13] and Chambadel [14] in 1957 for steady-flow power plants, while the systematical analysis combining thermodynamics with heat transfer for Carnot cycle was performed by Curzon and Ahlborn [15] in 1975 for reciprocating model, and FTT development was promoted by Berry’s group [4].

A large number of works have been performed for reciprocating (finite time) models [16,17,18,19,20,21,22,23,24,25] by applying FTT. While finite size is the major feature for steady-flow devices, such as closed gas rubine (Brayton cycle) power plants and steam (Rankine cycle) and organic Rankine cycle power plants, many scholars have performed FTT studies for various steady-flow cycles with the power output (POW), thermal efficiency (TEF), exergy efficiency, profit rate, and ecological function as the optimization goals, under the conditions of different losses and heat transfer laws [26,27,28,29,30,31,32,33,34,35,36,37,38,39,40,41,42,43,44,45,46,47,48,49,50,51].

Lenoir [52] first proposed the Lenoir cycle (LC) model in 1860. The simple LC consists of only three processes of constant-volume endothermic, adiabatic expansion, and constant-pressure exothermic; the LC is also called the triangular cycle. According to the cycle form, LC can be divided into steady-flow and reciprocating. Georgiou [53] first used classical thermodynamics to study the performances of simple, regenerated, and modified regenerated steady-flow Lenoir cycles (SFLCs).

Following on from [53], Shen et al. [54] applied FTT theory to optimize the POW and TEF characteristics of the endoreversible SFLC with only the loss of heat resistance, and they studied the influences of heat source temperature ratio and total heat conductance (HC) on cycle performance. Ahmadi et al. [55] used a genetic algorithm to carry out multiobjective optimization for endoreversible SFLC, and they obtained the optimal values of ecological performance coefficient and thermal economy under different temperature ratios.

In this paper, an irreversible SFLC model will be established on the basis of [54], while the cycle performance will be analyzed and optimized with the POW and TEF as objective functions, the optimal HC distributions of hot- and cold-side heat exchangers (HACHEX) of the cycle will be studied under different fixed total HCs, and the characteristic relationships between POW and TEF versus HC distribution are obtained. The effect of the internal irreversibility will be analyzed.

## 2. Cycle Model

**Figure 1:**
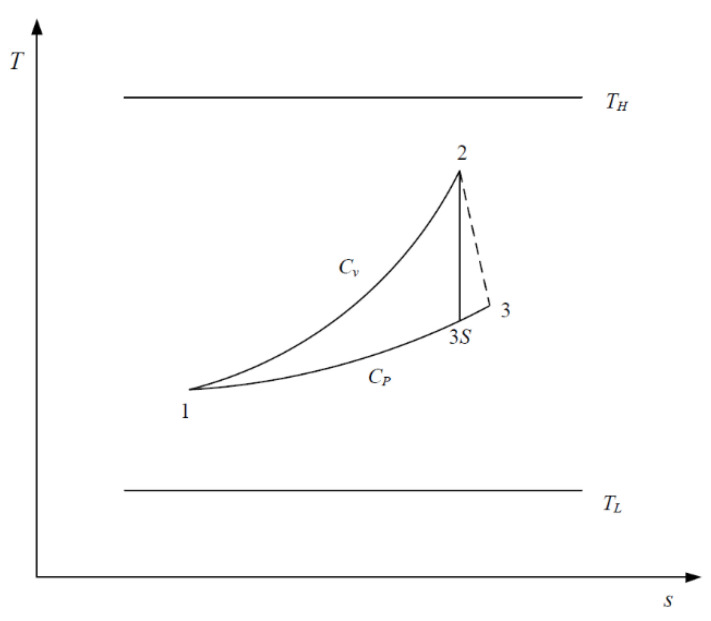
T−s diagram for the irreversible steady-flow Lenoir cycle (SFLC).

**Figure 2:**
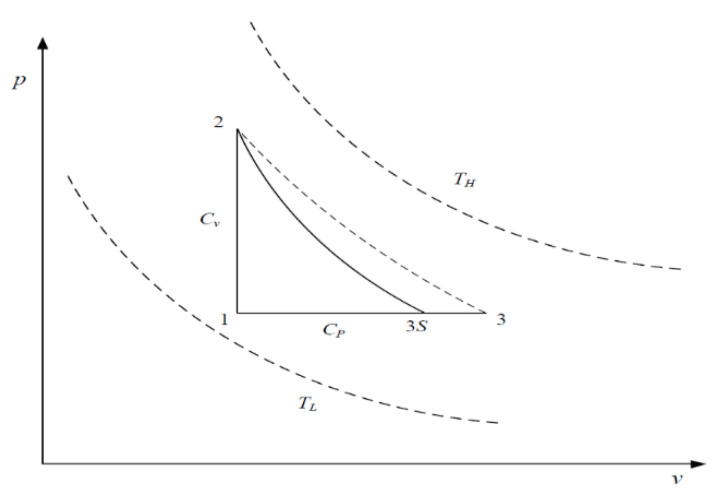
p−v diagram for the irreversible SFLC.

Figure 1 and Figure 2 show the T−s and p−v diagrams of the irreversible SFLC. As can be seen, 1→2 is the constant-volume endothermic process, 2→3 is the irreversible adiabatic expansion process (2→3S is the corresponding isentropic process), and 3→1 is the constant-pressure exothermic process. Assuming the cycle WF is an ideal gas, the entire cycle needs to be completed between the heat source ($T_{H}$) and heat sink ($T_{L}$).

In the actual work of the HEG, there are irreversible losses during compression and expansion processes; thus, the irreversible expansion efficiency $\eta_{E}$ is defined to describe the irreversible loss during the expansion process.

(1) $$\eta_{E}=\frac{T_{2}-T_{3}}{T_{2}-T_{3S}},$$

where $T_{i}$ (i=2,3,3S) is the corresponding state point temperature.

Assuming that the heat transfer between the WF and heat reservoir obeys the law of Newton heat transfer, according to the theory of the heat exchanger (HEX) and the ideal gas properties, the cycle heat absorbing and heat releasing rates are, respectively,

(2) $${\dot{Q}}_{1\to 2}=\dot{m}C_{v}E_{H}(T_{H}-T_{1})=\dot{m}C_{v}(T_{2}-T_{1}),$$

(3) $${\dot{Q}}_{3\to 1}=\dot{m}C_{P}E_{L}(T_{3}-T_{L})=\dot{m}C_{P}(T_{3}-T_{1}),$$

where $\dot{m}$ is the mass flow rate of the WF, $C_{v}$($C_{P}$) is the constant-volume (constant-pressure) SH ($C_{P}=kC_{v}$, *k* is the cycle SH ratio), and $E_{H}$($E_{L}$) is the effectiveness of hot-side (cold-side) HEX.

The relationships among the effectivenesses with the corresponding heat transfer unit numbers ($N_{H}$, $N_{L}$) and HCs ($U_{H}$, $U_{L}$) are as follows:

(4) $$N_{H}=U_{H}/(\dot{m}C_{v}),$$

(5) $$N_{L}=U_{L}/(\dot{m}kC_{v}),$$

(6) $$E_{H}=1-\exp(-N_{H}),$$

(7) $$E_{L}=1-\exp(-N_{L}).$$

## 3. Analysis and Discussion

### 3.1. Power and Thermal Efficiency Expressions

According to the second law of thermodynamics, after a cycle process, the total entropy change of the WF is equal to zero; thus, one finds

(8) $$C_{v}\ln(T_{2}/T_{1})-C_{P}\ln(T_{3S}/T_{1})=0.$$

From Equation (8), one obtains

(9) $$\frac{T_{2}}{T_{1}}=(\frac{T_{3S}}{T_{1}}{)}^{k}.$$

From Equations (2) and (3), one has

(10) $$T_{2}=E_{H}(T_{H}-T_{1})+T_{1},$$

(11) $$T_{3}=(E_{L}T_{L}-T_{1})/(E_{L}-1).$$

Combining Equations (1), (9), and (10) with Equation (11) yields

(12) $$T_{1}=\frac{E_{H}T_{H}(\eta_{E}-1)+(T_{1}-E_{L}T_{L})/(1-E_{L})}{\left\{(1-E_{H})(1-\eta_{E})+{\left\{[E_{H}T_{H}+(1-E_{H})T_{1}]/T_{1}\right\}}^{\frac{1}{k}}\eta_{E}\right\}}.$$

From Equations (2), (3) and (9)–(11), the POW and TEF expressions of the irreversible SFLC can be obtained as

(13) $$P={\dot{Q}}_{1\to 2}-{\dot{Q}}_{3\to 1}=\dot{m}C_{v}[E_{H}(T_{H}-T_{1})-\frac{kE_{L}(T_{1}-T_{L})}{1-E_{L}}],$$

(14) $$\eta =P/{\dot{Q}}_{1\to 2}=1-\frac{kE_{L}(T_{1}-T_{L})}{E_{H}(1-E_{L})(T_{H}-T_{1})}.$$

When $\eta_{E}=1$, Equation (12) simplifies to

(15) $$T_{1}-E_{L}T_{L}=(1-E_{L}){\left[E_{H}T_{H}+(1-E_{H})T_{1}\right]}^{\frac{1}{k}}T_{1}{}^{1-\frac{1}{k}}.$$

Equation (15) in this paper is consistent with Equation (15) in [54], where $T_{1}$ was obtained for the endoreversible SFLC. Combining Equations (13)–(15) and using the numerical solution method, the POW and TEF characteristics of the endoreversible SFLC in [54] can be obtained.

### 3.2. Case with Given Hot- and Cold-Side HCs

The working cycles of common four-branch HEGs, such as Carnot, Brayton, and Otto engines, can be roughly divided into four processes: compression, endothermic, expansion, and exothermic. Compared with these common four-stroke cycles, the biggest feature of the SFLC is the lack of a gas compression process, presenting a relatively rare three-branch cycle model.

When the hot- and cold-side HCs are constant, it can be seen from Equations (4)–(7) that the effectivenesses of the HACHEX which are directly related to each cycle state point temperature will be fixed values; as a result, the POW and TEF will also be fixed values.

### 3.3. Case with Variable Hot- and Cold-Side HCs When Total HC Is Given

When the HC changes, the POW and TEF of the cycle will also change; therefore, the HC can be optimized and the optimal POW and TEF can be obtained. Assuming the total HC is a constant,

(16) $$U_{L}+U_{H}=U_{T}.$$

Defining the HC distribution ratio as $u_{L}=\frac{U_{L}}{U_{T}}$($0<u_{L}<1$), from Equations (4)–(7), the effectivenesses of the HACHEX can be represented as

(17) $$E_{H}=1-\exp[-(1-u_{L})U_{T}/(\dot{m}C_{v})],$$

(18) $$E_{L}=1-\exp[-u_{L}U_{T}/(\dot{m}kC_{v})].$$

Combining Equations (12)–(14) and (17) with Equation (18) and using a numerical solution method, the characteristic relationships between POW and the hot- and cold-side HC distribution ratio, as well as between TEF and the hot- and cold-side HC distribution ratio, can be obtained.

## 4. Numerical Examples

It is assumed that the working fluid is air. Therefore, its constant-volume specific heat and specific heat ratio are $C_{v}=0.7165\;\mathrm{kJ}/(\mathrm{kg}\cdot K)$ and k=1.4. The turbine efficiency of the gas turbine is about $\eta_{E}=0.92$ in general. According to the [51,52,53,54,55], $\dot{m}=1.1165\;\mathrm{kg}/s$ and $T_{L}=320\;K$ were set.

**Figure 3:**
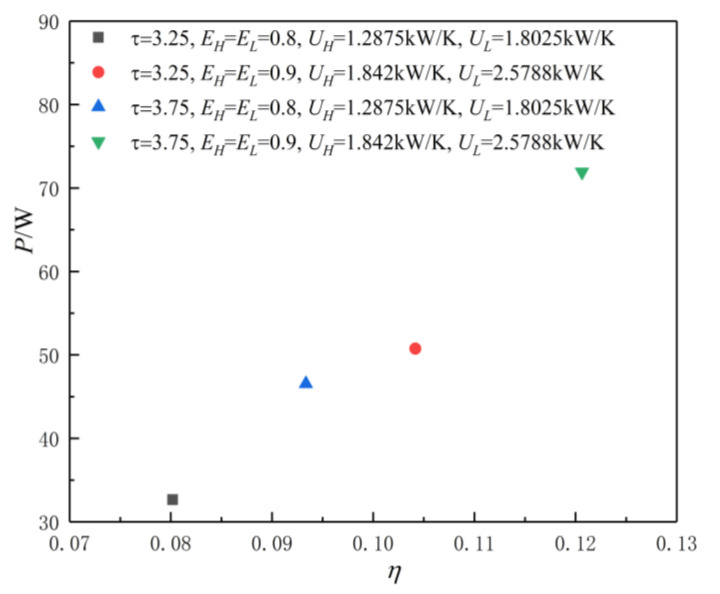
The power output (POW) and thermal efficiency (TEF) characteristics when the HCs of HACHEX are given.

**Figure 4:**
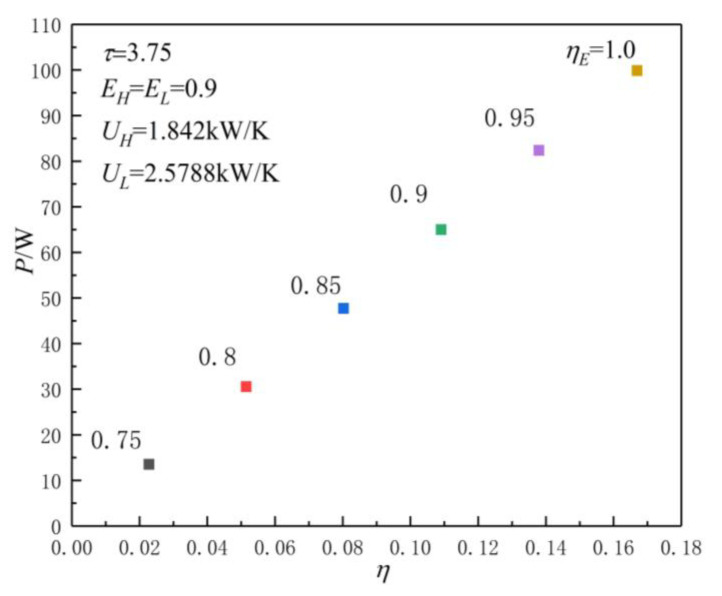
Effect of $\eta_{E}$ on P-η characteristics when the HCs of HACHEX are given.

Figure 3 shows the POW and TEF characteristics when the HCs of the HACHEX and temperature ratio are different values. When the HCs and temperature ratio are fixed values, the effectivenesses of the HEX are fixed values, and the corresponding POW and TEF are also fixed values. The POW and TEF characteristics are reflected in the graph as a point. As can be seen, when τ($\tau =T_{H}/T_{L}$) and the HCs of the HEXs increase, the corresponding POW and TEF increase. Figure 4 shows the influence of $\eta_{E}$ on P-η characteristics when the HCs of HACHEX and temperature ratio are given. As can be seen, with the increase in $\eta_{E}$ (the decrease of irreversible loss), the corresponding P and η increase.

**Figure 5:**
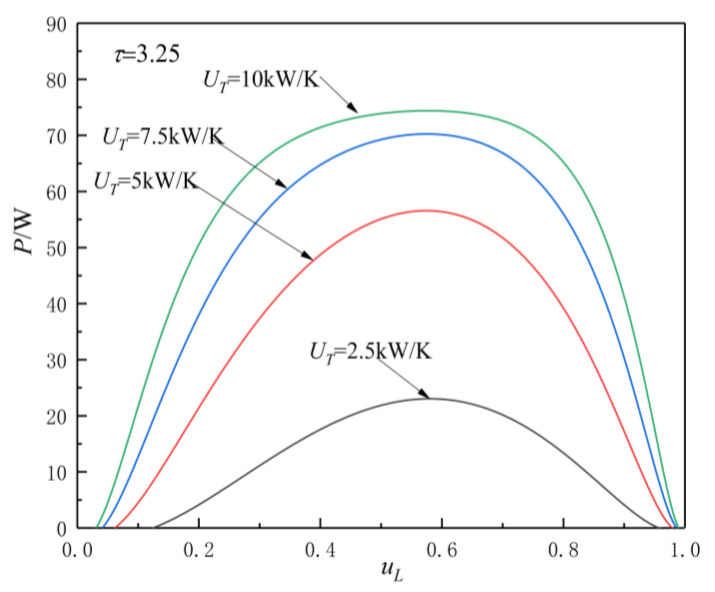
Effect of $U_{T}$ on $P-u_{L}$ characteristics when τ=3.25.

**Figure 6:**
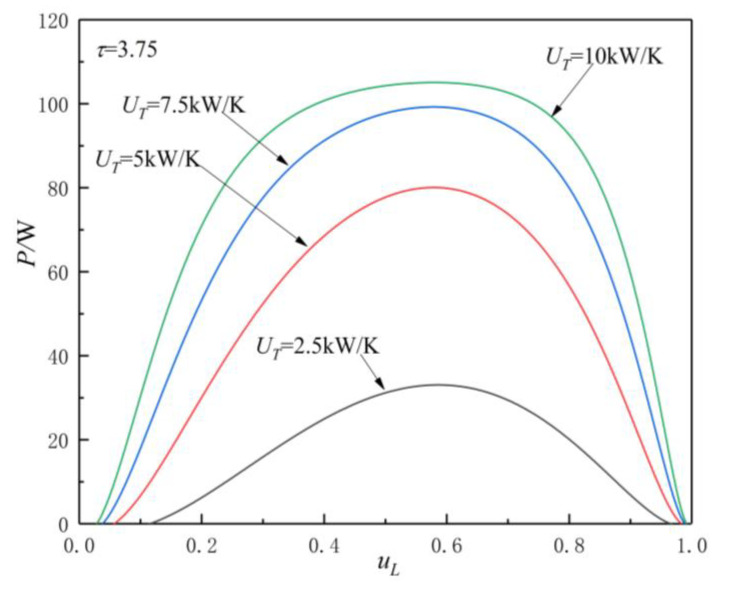
Effect of $U_{T}$ on $P-u_{L}$ characteristics when τ=3.75.

**Figure 7:**
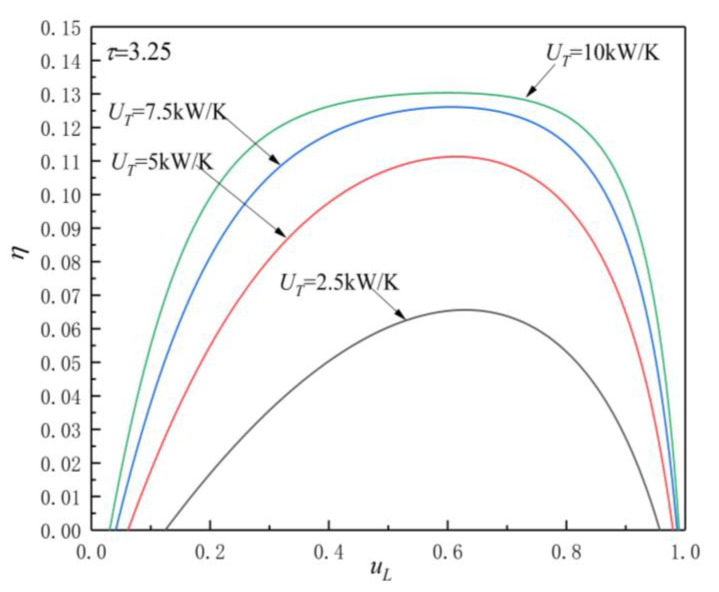
Effect of $U_{T}$ on $\eta -u_{L}$ characteristics when τ=3.25.

**Figure 8:**
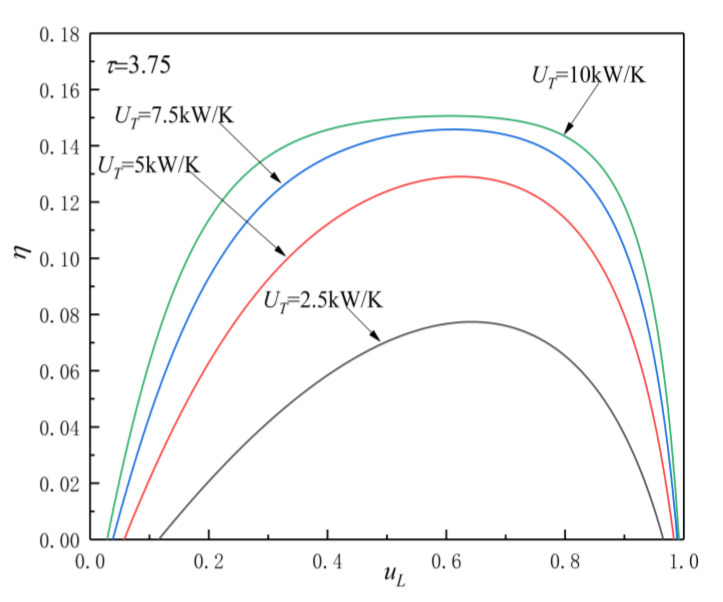
Effect of $U_{T}$ on $\eta -u_{L}$ characteristics when τ=3.75.

Figure 5, Figure 6, Figure 7 and Figure 8 show the influences of $U_{T}$ on the $P-u_{L}$ and $\eta -u_{L}$ characteristics when τ=3.25 and τ=3.75. The relationship curves of $P-u_{L}$ and $\eta -u_{L}$ are parabolic-like changes. With the increase in $u_{L}$, the corresponding POW and TEF first increase and then decrease, and there are optimal HC distribution values $u_{L_{P}(opt)}$ and $u_{L_{\eta }(opt)}$, which lead to POW and TEF reaching their maximum values $P_{\max}$ and $\eta_{\max}$.

Figure 5 and Figure 6 show the influence of $U_{T}$ on $P-u_{L}$ characteristics when τ=3.25 and τ=3.75. As can be seen, with the increase in $U_{T}$, $P_{\max}$ increases and $u_{L_{P}(opt)}$ decreases. When $U_{T}$ is 2.5, 5, 7.5, and 10 kW/K and τ=3.25, the corresponding $P_{\max}$ is 23.04, 56.58, 70.25, and 74.39 W, while $u_{L_{P}(opt)}$ is 0.58, 0.575, 0.574, and 0.573, respectively. When $U_{T}$ changes from 2.5 to 10 kW/K, the corresponding $P_{\max}$ increases by about 222.9%, while the $u_{L_{P}(opt)}$ decreases by about 1.21%. When $U_{T}$ is 2.5, 5, 7.5, and 10 kW/K and τ=3.75, the corresponding $P_{\max}$ is 33.06, 80.06, 90.24, and 105.06 W, while $u_{L_{P}(opt)}$ is 0.586, 0.579, 0.5785, and 0.5782, respectively. When $U_{T}$ changes from 2.5 to 10 kW/K, the corresponding $P_{\max}$ increases by about 217.8%, while the $u_{L_{P}(opt)}$ decreases by about 1.33%.

Figure 7 and Figure 8 show the influence of $U_{T}$ on $\eta -u_{L}$ characteristics when τ=3.25 and τ=3.75. As can be seen, with the increase in $U_{T}$, $\eta_{\max}$ increases and $u_{L_{\eta }(opt)}$ decreases. When $U_{T}$ is 2.5, 5, 7.5, and 10 kW/K and τ=3.25, the corresponding $\eta_{\max}$ is 0.066, 0.111, 0.126, and 0.1303, while $u_{L_{\eta }(opt)}$ is 0.629, 0.614, 0.605, and 0.6, respectively. When $U_{T}$ changes from 2.5 to 10 kW/K, the corresponding $\eta_{\max}$ increases by about 97.4%, while $u_{L_{P}(opt)}$ decreases by about 4.61%. When $U_{T}$ is 2.5, 5, 7.5, and 10 kW/K and τ=3.75, the corresponding $\eta_{\max}$ is 0.0774, 0.129, 0.1458, and 0.1506, while $u_{L_{\eta }(opt)}$ is 0.644, 0.624, 0.608, and 0.606, respectively. When $U_{T}$ changes from 2.5 to 10 kW/K, the corresponding $\eta_{\max}$ increases by about 94.6%, while $u_{L_{P}(opt)}$ decreases by about 5.9%.

From Figure 5, Figure 6, Figure 7 and Figure 8 and Equations (12)–(14), (17), and (18), one can see that, when τ is given, the POW and TEF are mainly affected by the total HC; with the increase in $U_{T}$, the $P_{\max}$ and $\eta_{\max}$ increase. When the total HC is small, the corresponding $P_{\max}$ and $\eta_{\max}$ change more significantly. When the total HC is large, the corresponding $P_{\max}$ and $\eta_{\max}$ change little. When $U_{T}$ is given, with the increase in τ, the $u_{L_{P}(opt)}$ and $u_{L_{\eta }(opt)}$ increase. When τ and $U_{T}$ are given, the corresponding $u_{L_{\eta }(opt)}>u_{L_{P}(opt)}$.

**Figure 9:**
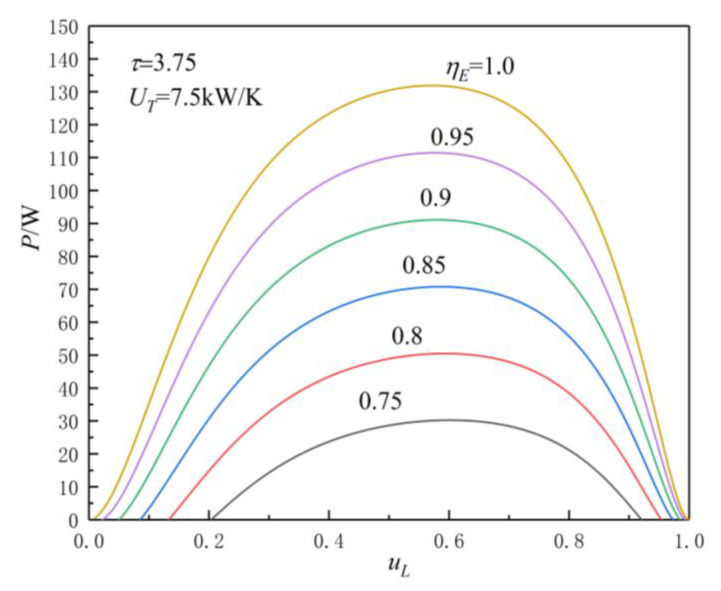
Effect of $\eta_{E}$ on $P-u_{L}$ characteristics.

**Figure 10:**
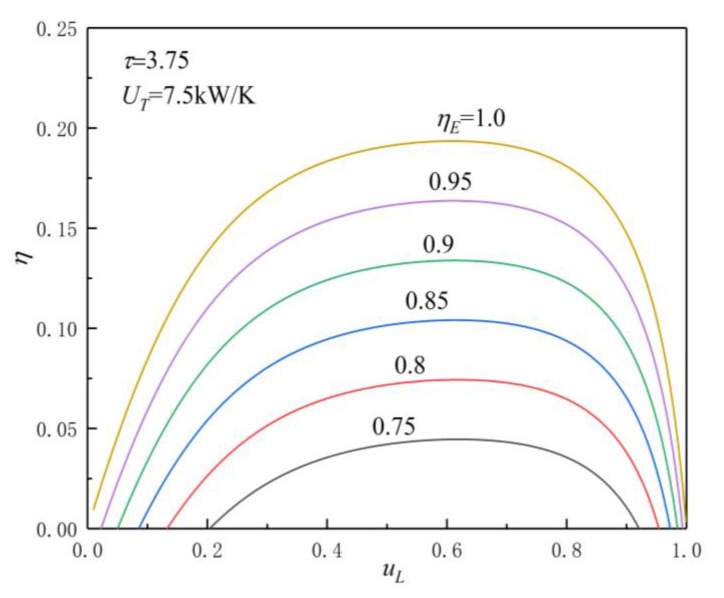
Effect of $\eta_{E}$ on $\eta -u_{L}$ characteristics.

Figure 9 and Figure 10 show the influences of $\eta_{E}$ on $P-u_{L}$ and $\eta -u_{L}$ characteristics when τ=3.75 and $U_{T}=7.5\;\mathrm{kW}/K$. As can be seen, when τ=3.75 and $U_{T}=7.5\;\mathrm{kW}/K$, with the increase in $\eta_{E}$ (the decrease in irreversible loss), the $P_{\max}$ and $\eta_{\max}$ increase, while the corresponding $u_{L_{P}(opt)}$ and $u_{L_{\eta }(opt)}$ decrease. When $\eta_{E}$ is 0.75, 0.8, 0.85, 0.9, 0.95, and 1.0, the corresponding $P_{\max}$ is 30.2431, 50.4808, 70.7674, 91.0982, 111.4719, and 131.8876, $\eta_{\max}$ is 0.0445, 0.0743, 0.1041, 0.1339, 0.1637, and 0.1935, $u_{L_{P}(opt)}$ is 0.601, 0. 593, 0.586, 0.581, 0.576, and 0.572, and $u_{L_{\eta }(opt)}$ is 0.619, 0.617, 0.615, 0.613, 0.611, and 0.609, respectively. When $\eta_{E}$ changes from 0.75 to 1.0, the corresponding $P_{\max}$ increases by about 336.1%, $\eta_{\max}$ increases by about 334.8%, $u_{L_{P}(opt)}$, and $u_{L_{\eta }(opt)}$ decreases by about 4.83% and 1.62%, respectively.

## 5. Conclusions

In this paper, an irreversible SFLC model is established on the basis of [54], while the POW and TEF characteristics of the irreversible SFLC were studied using FTT theory, and the influences of τ, $U_{T}$ and $\eta_{E}$ on $P_{\max}$, $\eta_{\max}$, $u_{L_{P}(opt)}$, and $u_{L_{\eta }(opt)}$ were analyzed. The main conclusions are as follows:

(1) When the HCs are constants, the corresponding POW and TEF are fixed values. When τ and the HCs of the HEXs increase, the corresponding POW and TEF increase. When τ and HCs of the HEXs are constants, with the increase in $\eta_{E}$ (the decrease in irreversible loss), the corresponding P and η increase.
(2) When the distribution of HCs can be optimized, the relationships of $P-u_{L}$ and $\eta -u_{L}$ are parabolic-like ones.
(3) When $U_{T}$ is given, with the increase in τ, $P_{\max}$, $\eta_{\max}$, $u_{L_{P}(opt)}$, and $u_{L_{\eta }(opt)}$ increase.
(4) When τ is given, with the increase in $U_{T}$, $P_{\max}$ and $\eta_{\max}$ increase, while $u_{L_{P}(opt)}$ and $u_{L_{\eta }(opt)}$ decrease. When τ and $U_{T}$ are given, the corresponding $u_{L_{\eta }(opt)}$ is bigger than $u_{L_{P}(opt)}$.
(5) When τ=3.75 and $U_{T}=7.5\mathrm{kW}/K$, with the increase in $\eta_{E}$, $P_{\max}$ and $\eta_{\max}$ increase, while the corresponding $u_{L_{P}(opt)}$ and $u_{L_{\eta }(opt)}$ decrease.

## Nomenclature

$C_{P}$	Specific heat at constant pressure (kJ/(kg⋅K))
$C_{v}$	Specific heat at constant volume (kJ/(kg⋅K))
E	Effectiveness of heat exchanger
k	Specific heat ratio (-)
$\dot{m}$	Mass flow rate of the working fluid (kg/s)
N	Number of heat transfer units
P	Cycle power (W)
$\dot{Q}$	Quantity of heat transfer rate (W)
T	Temperature (K)
U	Heat conductance (kW/K)
$U_{T}$	Total heat conductance (kW/K)
u	Heat conductance distribution
Greek symbols	
τ	Temperature ratio
η	Cycle thermal efficiency
Subscripts	
H	Hot-side
L	Cold-side
max	Maximum value
opt	Optimal
P	Maximum power point
η	Maximum thermal efficiency point
1-3,3S	Cycle state points

## Abbreviations

FTT	Finite time thermodynamic
HACHEX	Hot- and cold-side heat exchangers
HC	Heat conductance
HEG	Heat engine
HEX	Heat exchanger
LC	Lenoir cycle
POW	Power output
SFLC	Steady flow Lenoir cycle
SH	Specific heat
TEF	Thermal efficiency
WF	Working fluid
